# Conventional type-1 DC density is associated with checkpoint inhibitor response across multiple types of cancer

**DOI:** 10.1172/JCI200987

**Published:** 2026-05-01

**Authors:** Alvaro Lopez-Janeiro, José González-Gomariz, Fadi Issa, Joanna Hester, Angelo Porciuncula, Alvaro Teijeira, Carlos Luri-Rey, David Ruiz-Guillamon, Jose Luis Perez-Gracia, Elisabeth Perez-Ruiz, Isabel Barragan, Salvador Martín-Algarra, Miguel F. Sanmamed, Ignacio Ortego, Maria E. Rodriguez-Ruiz, Raluca Alexandru, Inmaculada Rodriguez, Saioa Arrieta-Aranzueque, David Rimm, Thazin Aung, Kurt A. Schalper, Carlos E. de Andrea, Ignacio Melero

**Affiliations:** 1 Department of Pathology, Clinica Universidad de Navarra. Pamplona, Navarra, Spain.; 2 Institute of Data Science and Artificial Intelligence (DATAI), Universidad de Navarra, Pamplona, Spain.; 3 TECNUN School of Engineering, Universidad de Navarra, San Sebastian, Spain.; 4 DigiMedLab, Cancer Center Clínica Universidad de Navarra (CCUN), Pamplona, Spain.; 5 Immunology and Immunotherapy Program, Cima Universidad de Navarra, Pamplona, Spain.; 6 Nuffield Department of Surgical Sciences, University of Oxford, Oxford, United Kingdom.; 7 Department of Pathology, Yale University School of Medicine, New Haven, Connecticut, USA.; 8 Centro de Investigación Biomédica en Red de Cáncer (CIBERONC), Madrid, Spain.; 9 Instituto de Investigación Sanitaria de Navarra (IDISNA), Pamplona, Spain.; 10 Department of Oncology, Cancer Center Clinica Universidad de Navarra, Pamplona, Spain.; 11 Group of Translational Research in Cancer Immunotherapy and Epigenetics (B-05), Medical Oncology Department, Hospital Regional Universitario de Malaga, Instituto de Investigación Biomédica de Málaga y Plataforma en Nanomedicina–IBIMA Plataforma BIONAND, Malaga, Spain.; 12 Group of Translational Research in Cancer Immunotherapy and Epigenetics (B-05), Medical Oncology Unit of Virgen de la Victoria Hospital, Instituto de Investigación Biomédica de Málaga y Plataforma en Nanomedicina–IBIMA Plataforma BIONAND, 29010, Malaga, Spain.; 13 Group of Pharmacoepigenetics, Department of Physiology and Pharmacology, Karolinska Institute, Stockholm, Sweden.; 14 Department of Radiation Oncology, Clínica Universidad de Navarra, Pamplona, Spain.

**Keywords:** Immunology, Oncology, Cancer immunotherapy, Dendritic cells

## Abstract

Conventional type-1 dendritic cells (cDC1) are the main mediators of crosspresentation of tumor antigens to CD8^+^ T cells and provide a context of costimulatory molecules and cytokines that lead to cytotoxic T lymphocyte (CTL) responses. We analyzed bulk RNA sequences from 7 key clinical trials testing checkpoint inhibitors across multiple cancer types. cDC1- and CD8-associated gene signatures were analyzed. Multiplex tissue immunofluorescence was used to quantify cDC1 in melanoma, urothelial cancer, and non-small-cell lung cancer (NSCLC) samples and assess cDC1 tissue neighborhoods. Melanoma samples were studied with Xenium spatial transcriptomics (ST) and one series of NSCLC was analyzed using GeoMX-DSP. Strong associations across tumor types were found between cDC1 and CD8^+^ T cell transcripts with clinical outcomes. As mechanistically expected, transcripts for the CCL4 and CCL5 chemokines and the growth factor FLT3-L showed associations with cDC1 abundance. Tissue immunofluorescence showed a strong correlation of cDC1 and CD8^+^ T cell infiltration with clinical benefit upon treatment with checkpoint inhibitors (CPIs). Moreover, short distance between cDC1 and CD8^+^ T cells was found to define tissue niches associated with favorable outcomes. ST revealed recent T cell activation within immune cDC1-rich niches. cDC1 abundance, which determines CD8^+^ T lymphocyte density and activation in tumor tissues across cancer types, is strongly associated with clinical response to CPI-based immunotherapies.

## Abstract 2

Dendritic cells, a kind of cells involved in lymphocyte activation, are pivotal to benefit from current immunotherapies.

## Introduction

Antigen crosspresentation and crosspriming are critical functions for the activation of CD8^+^ T cell immune responses (1, 2). It is well established that these functions are mainly mediated by a dendritic cell subtype named conventional type-1 DC (cDC1), whose independent identity and ontogenic lineage is well documented in mice and humans (3, 4).

This capability to activate CD8^+^ T cells is dependent on the almost unique ability of cDC1s to redirect endocytosed antigens to the MHC Class-I presentation pathway (1, 5). Moreover, upon activation, cDC1 may acquire optimal surface expression of costimulatory ligands and cytokines to prime and sustain CD8^+^ T cell activation in mice and humans (1, 6–8).

Interestingly, the BATF3 transcription factor is almost exclusive of this DC subset and is critical for the ontogeny differentiation of cDC1, while other DC subsets are preserved in BATF3-deficient mice (9). Lack of cDC1 in BATF3-KO mice results in a complete inability to promote CD8^+^ T cell fitness and functionality against viruses or immunogenic tumors (10). These results have been confirmed in other engineered mouse strains with alterations in the promoter of IRF8 that are also completely devoid of cDC1 (11).

Experiments using mice defective in cDC1 cells or transgenic mice selectively depletable of cDC1 cells, clearly demonstrated that these professional antigen-presenting cells were an absolute requirement for efficacy of immunotherapy with checkpoint inhibitors (12, 13). Additional evidence in mouse tumors indicates that even small numbers of tumor-infiltrating cDC1 organize and orchestrate CD8^+^ T cell responses in tumors in conjunction with NK cells (14, 15).

Pioneering work by S. Spranger and T. Gajewski evaluating TCGA and other datasets showed that, in human melanoma, the transcripts defining the presence of cDC1 in tumor tissue were associated with CD8^+^ T cell density much more strongly than tumor mutational burden. Moreover, the spatial density of cDC1 cells in tumor has a prognostic value (16).

Small series of melanoma and breast cancer samples have also been studied for the density of cDC1 cells and their positive association with response to checkpoint inhibitor therapy, as revealed using XCR1, CLEC9A, and BATF3 as cDC1 markers (17–20). Prostaglandins and perhaps other immunosuppressive factors seem to locally reduce cDC1 density in the tumor microenvironment and limit response to checkpoint inhibitors (21, 22).

The group of Miriam Merad has further advanced this field upon the study of a series of patients undergoing neoadjuvant treatment with nivolumab for surgically resectable hepatocellular carcinoma (HCC). This study showed the importance of the presence and density of activated cDC1 in close proximity to CD4^+^ and CD8^+^ T cells forming triads in the tumors (7). Functional interplays between these cell subtypes and also with NK cells and macrophages are postulated to be favorable to mount cytotoxic antitumor immune responses (23) and organize tertiary lymphoid structures (24).

In this study, we sought to generalize the concept that cDC1 presence/abundance is associated with higher CD8^+^ T cell densities in baseline (pretreatment) samples and is determinant of favorable clinical outcomes upon treatment with PD-(L)1 checkpoint inhibitors in large series of malignant tumor tissue samples (25, 26). Making use of the heterogeneity of the tumor microenvironment (27), we found much stronger statistical associations if considering the cDC1-to-CD8 distances and their spatial organization in tissular niches that coordinate key spatial transcriptional programs in the tumor microenvironment.

## Results

### Gene signatures of cDC1 cells are associated with clinical benefit from checkpoint inhibitors across clinical trials.

**Figure 1:**
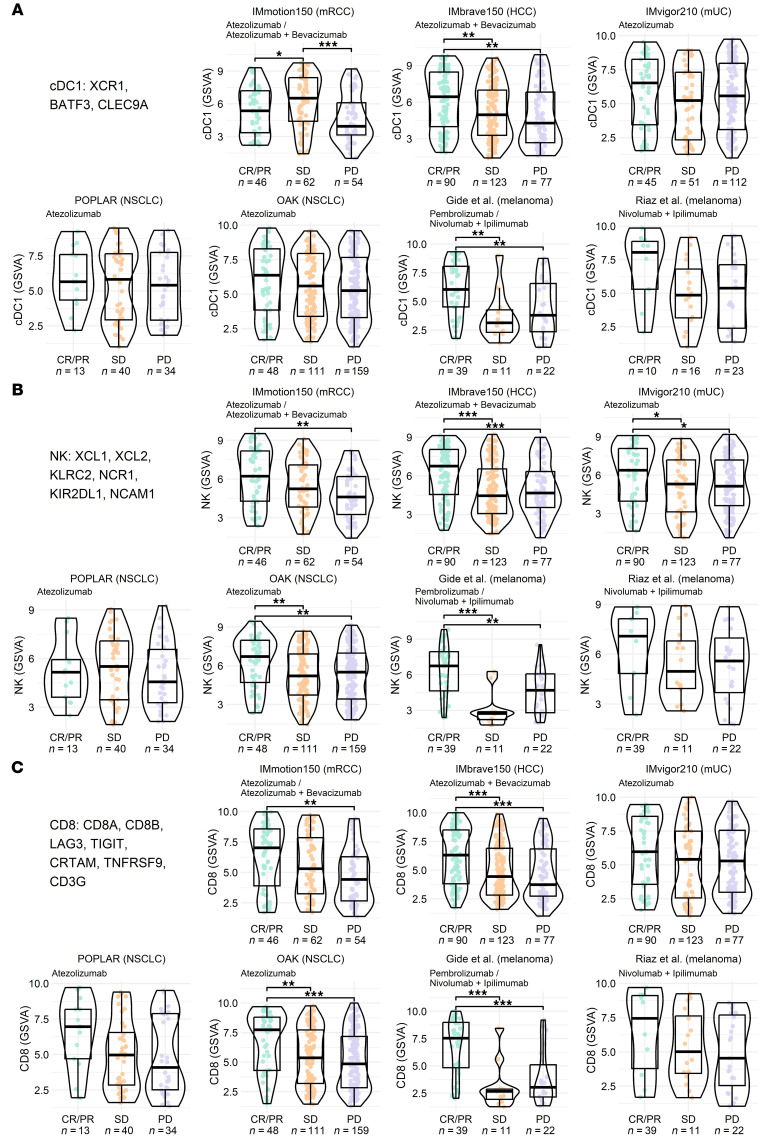
Clinical benefit from checkpoint inhibitors is associated with a cDC1 gene signature across checkpoint inhibitor clinical trials for multiple cancer histologies. (**A**) Analyses of the gene signature of cDC1 in the tumor biopsy RNA-seq datasets based on XCR1, BATF3, and CLEC9A transcripts from patients participating in the indicated clinical trials and receiving the specified immunotherapy treatments who were classified according to clinical benefit. CR/PR, Complete response or Partial response; SD, Stable disease; PD, Progressive disease. (**B** and **C**) similar data analyzed for an NK-cell signature (**B**) and a signature denoting CD8^+^ T lymphocytes (**C**) whose genes are listed. *P < 0.05, **P < 0.01, ***P < 0.001; Wilcoxon significance tests.

The abundance of cDC1 cells in tumor tissue can be inferred with a 3 gene–based signature, including BATF3, XCR1, and CLEC9A (15, 22). We gained access to bulk RNA-seq datasets from before treatment, as well as pretreatment tumor samples from 7 key clinical trials sponsored by industry, which study checkpoint inhibitors for renal cell carcinoma (RCC), HCC, NSCLC, urothelial carcinoma, and melanoma, as described in Methods. Figure 1A shows that across the clinical trial series, cDC1 transcripts in the arms of patients treated with checkpoint inhibitors were associated with better clinical outcomes in terms of objective response or disease stabilization. Clinical benefit was also associated with gene signatures that estimate the abundance of NK or CD8^+^ T cells defined using our refined gene signatures (see Methods section) (Figure 1, B and C).

**Figure 2:**
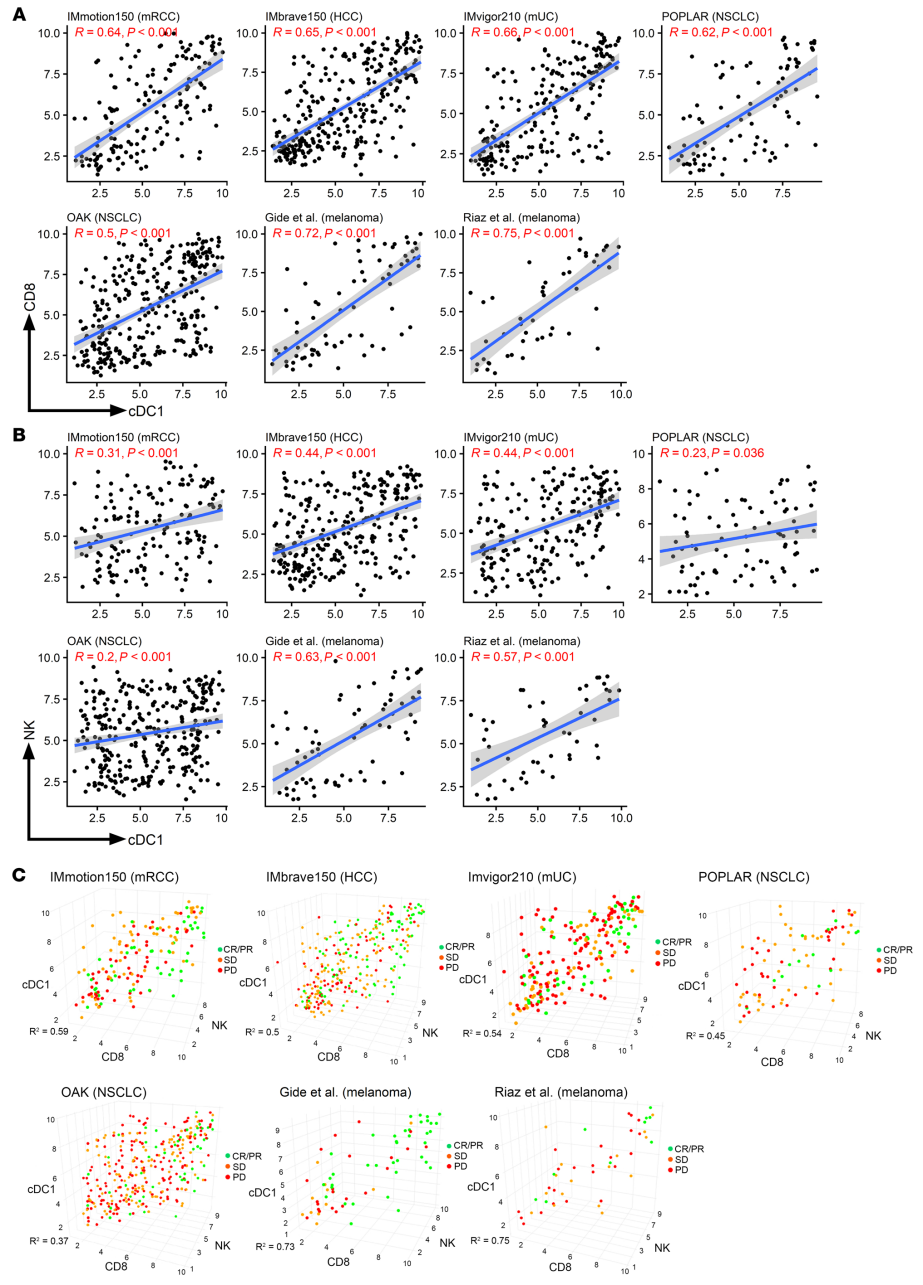
The cDC1 gene signature correlates with CD8 and NK signatures. (**A** and **B**) Gene expression datasets from the clinical trials in Figure 1 were studied for the association of the gene signatures of cDC1 with the gene signatures for CD8^+^ T cells (**A**) and NK cells (**B**). (**C**) Shows 3D representations indicating that signatures were mutually associated. R represents the Pearson correlation index and *P* values according to linear regression. Shaded area confidence intervals for each linear regression line are shown. Color coded dots in **C** indicate clinical benefit.

Importantly, across all the tumor types included in these trials, we observed a very strong correlation between the cDC1 and CD8 signatures. The correlation between cDC1- and NK-denoting transcript signatures was weaker (Figure 2, A and B). 3D representations indicate that some kind of triple association among these 3 cell types is observable in most cases (Figure 2C) and is generally associated with better clinical responses.

In contrast, the association with clinical benefit was not observed in all of the 4 corresponding control arms of the trials treated with TKIs or taxanes (Supplemental Figure 1, A–C; supplemental material available online with this article; https://doi.org/10.1172/JCI200987DS1), indicating more predictive than prognostic value, even though the positive correlation between CD8 and cDC1 transcripts was preserved (Supplemental Figure 1, D and E). Moreover, CD274 (PD-L1) transcripts showed variable association with clinical response across immunotherapy trial arms (Supplemental Figure 2).

### cDC1 abundance correlates with transcripts associated with chemoattraction of the cDC1 subset.

**Figure 3:**
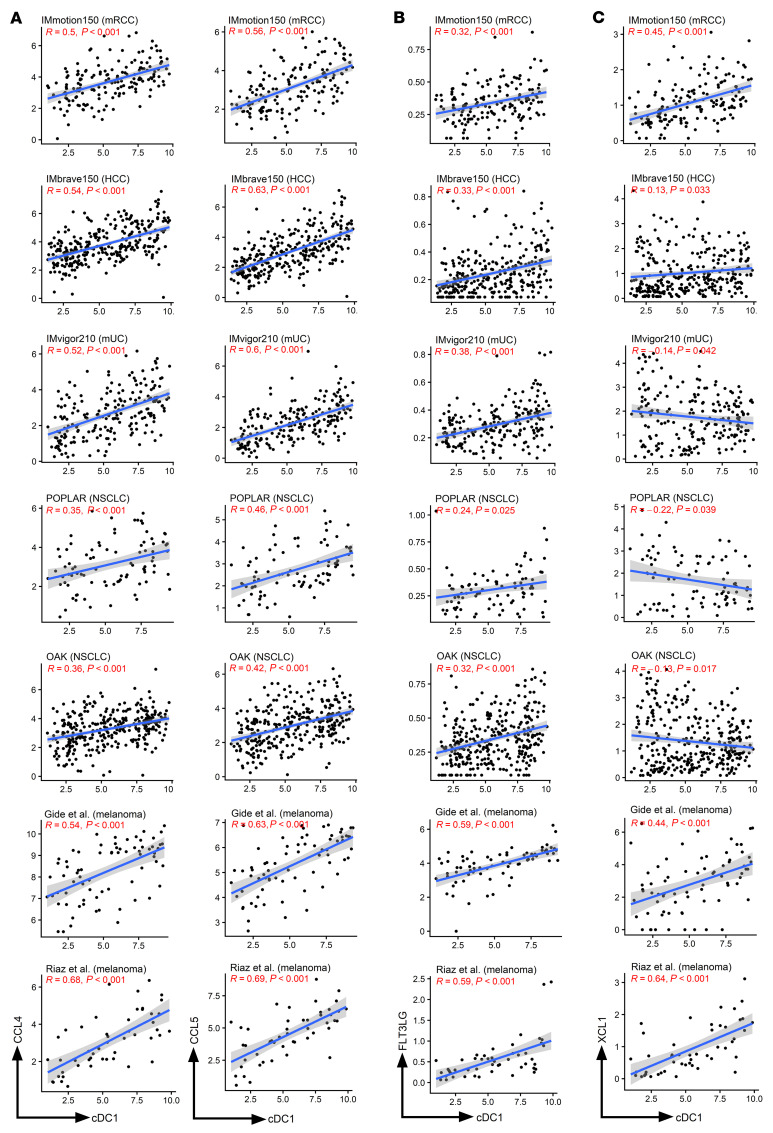
cDC1 infiltration denoted by its gene signature is associated with transcripts of the CCL4 and CCL5 chemokines. (**A**) Association of cDC1 gene signatures with the relative expression of the transcripts encoding the CCL4 and CCL5 chemokines in the indicated clinical trial datasets. (**B**) Similar correlation but with the expression of the FLT3LG transcript. (**C**) Lack of association of the cDC1 signature with the XCL1 chemokine transcript. R represents the Pearson correlation index and *P* values according to linear regression. Shaded area confidence intervals for each linear regression line are shown.

cDC1s are known to be selectively chemoattracted by CCL4 and CCL5 in a manner that has been shown in mice to be associated to immunotherapy outcomes (28, 29). Indeed, we observed across the studied trials’ RNA-seq datasets a strong positive correlation of the transcripts of these chemokines with the cDC1 gene signature (Figure 3A). Furthermore, the growth factor FLT-3L known to be critical for cDC1 differentiation, attraction, and maintenance (1, 30) was also correlated with cDC1-transcript abundance in the tumor tissue microenvironment (Figure 3B). However, the association with XCL1 chemokine transcript, which is produced by CD8^+^ T lymphocytes and NK cells to meet cDC1 (31), showed a variable and weaker association across trials. We even observed negative associations of XCL1 and cDC1 signature in 3 of the studied trials (Figure 3C). Importantly, in the corresponding chemotherapy and TKI control arms, the associations of cDC1 signatures and the transcripts for their chemoattracting chemokines were preserved (Supplemental Figure 3).

### cDC1, CD8, and CD4 densities measured by Multiplex tissue immunofluorescence are associated with clinical benefit in patients receiving checkpoint inhibitors.

**Figure 4:**
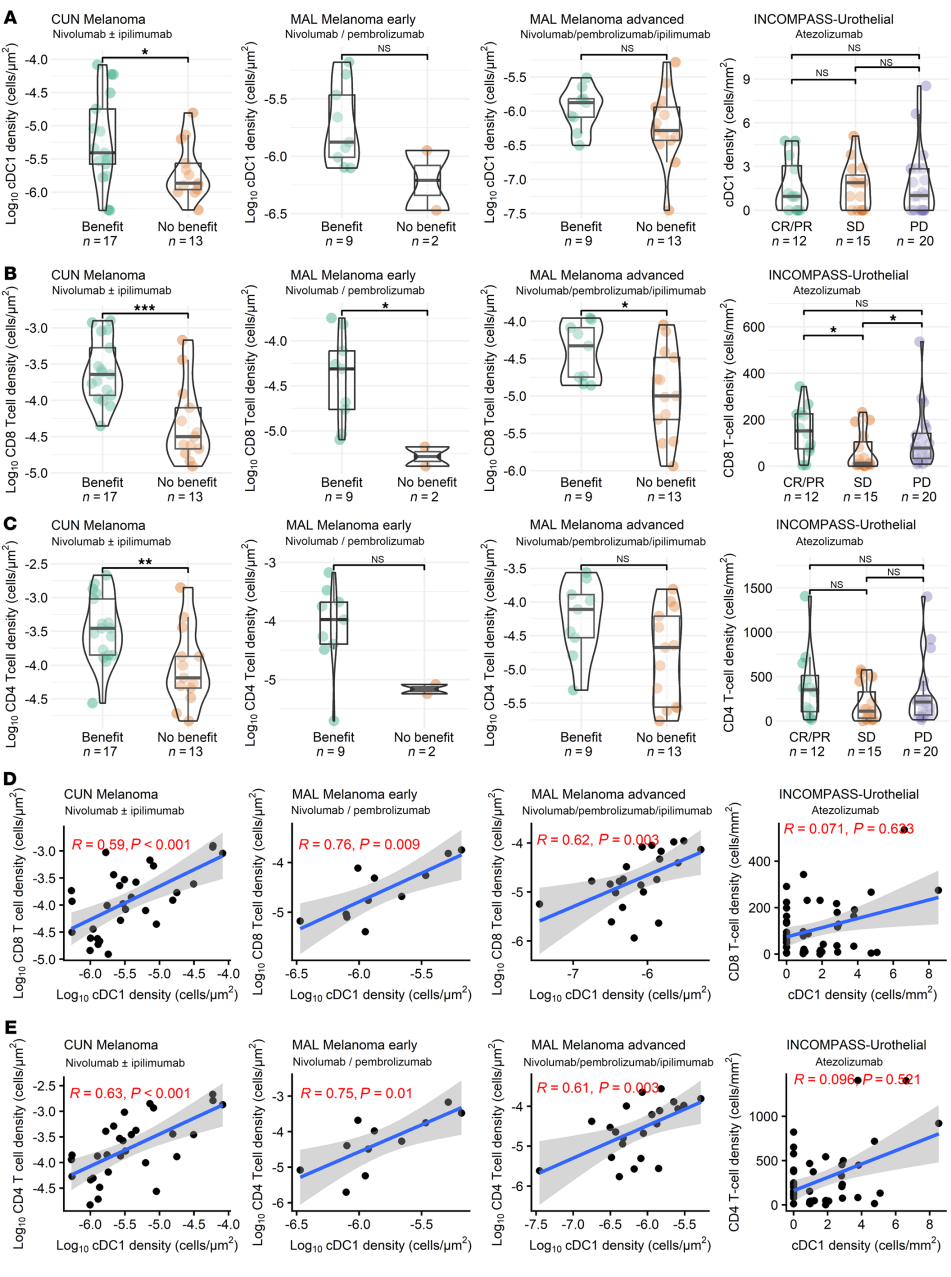
cDC1 cell density estimated by multiplex tissue immunofluorescence is associated with clinical benefit in 4 independent cohorts of patients encompassing melanoma and urothelial carcinoma. (**A**–**C**) Multiple tissue immunofluorescence assessments in 4 series of pretreatment biopsies from patients treated as indicated in independent series of patients suffering melanoma (CUN Melanoma, MAL melanoma early, MAL melanoma advanced) or urothelial carcinoma (Incompass trial). These included patients treated with adjuvant checkpoint inhibitors (MAL Melanoma early) or at metastatic stage (CUN Melanoma cohort), metastatic melanoma (MAL melanoma-advanced), and metastatic bladder cancer (Incompass). (**A**–**C**) Associations of the estimated densities of BATF3^+^ cDC1 cells, CD8^+^ T cells, CD4^+^ T cells with clinical outcome are shown with the corresponding statistical associations assessed: *P < 0.05, **P < 0.01, ***P < 0.001; Wilcoxon tests. **D** and **E** Show the correlation between cDC1 tumor tissue density and CD8^+^ or CD4^+^ T cell populations (Spearman rank correlation with associated *P* values). Shaded area confidence intervals for each linear regression line are shown in **D** and **E**.

We studied, using multiplex immunofluorescence (mIF) antibody panels, the density of cDC1 and T lymphocytes in pretreatment tissue samples from 3 distinct and independent collections of melanoma samples and 1 collection of advanced urothelial carcinoma samples. The CUN series included patients treated with anti-PD1 or anti-CTLA4 for advanced melanoma. An early melanoma cohort treated with adjuvant immunotherapy and followed for relapse for 12 months (MAL Melanoma early) and a confirmatory metastatic melanoma cohort (MAL Melanoma advanced) treated with anti-PD1 or anti-PD1 + anti-CTLA4. Moreover, we analyzed a nonoperable advanced/metastatic urothelial carcinoma cohort from a clinical trial testing anti-PDL1 (Atezolizumab). Overall, we identified 10.4 × 10^6^ cells across all tumor images, including 12,913 cDC1 cells, 506,365 CD4^+^ T cells and 300,330 CD8^+^ T cells. In accordance with our bulk transcriptomic analysis, cDC1 density was associated with clinical benefit in the 4 series of tissue samples, albeit this association was imperfect in some cases where tumor heterogeneity was noted (Figure 4A and Supplemental Figure 4A). This heterogeneity was partially explained by the low abundance of cDC1 cells, as BATF3 positive cDC1 cells were relatively scarce in most samples, representing less than 1% of all identified cells (Supplemental figure 4B). Moreover, there was a strong correlation between clinical benefit and CD8 (Figure 4B) and CD4 (Figure 4C) T cell densities. As expected, CD8 density showed the strongest association (25). Figure 4, D and E shows a strong association of cDC1 density with the density of CD8^+^ and CD4^+^ T cells in these samples.

Moreover, we observed a similar association in a validation NSCLC cohort from the Yale-New Haven Hospital in which we found substantial interpatient heterogeneity in the density of cDC1 cells (Supplemental Figure 5A). The abundance of cDC1 cells, in this case measured by XCR1/CD11c, was positively correlated with overall survival in immunotherapy-treated lung carcinoma samples, albeit without reaching statistical significance (Log-Rank *P* value = 0.18, Supplemental Figure 5B). Furthermore, we also observed an association of cDC1 density with improved survival according to cDC1 density deciles (HR per decile increase = 0.94, *P* value = 0.11).

### Spatial interactions between cDC1 and CD8^+^ T cells are associated with better clinical outcomes upon treatment with checkpoint inhibitors.

**Figure 5:**
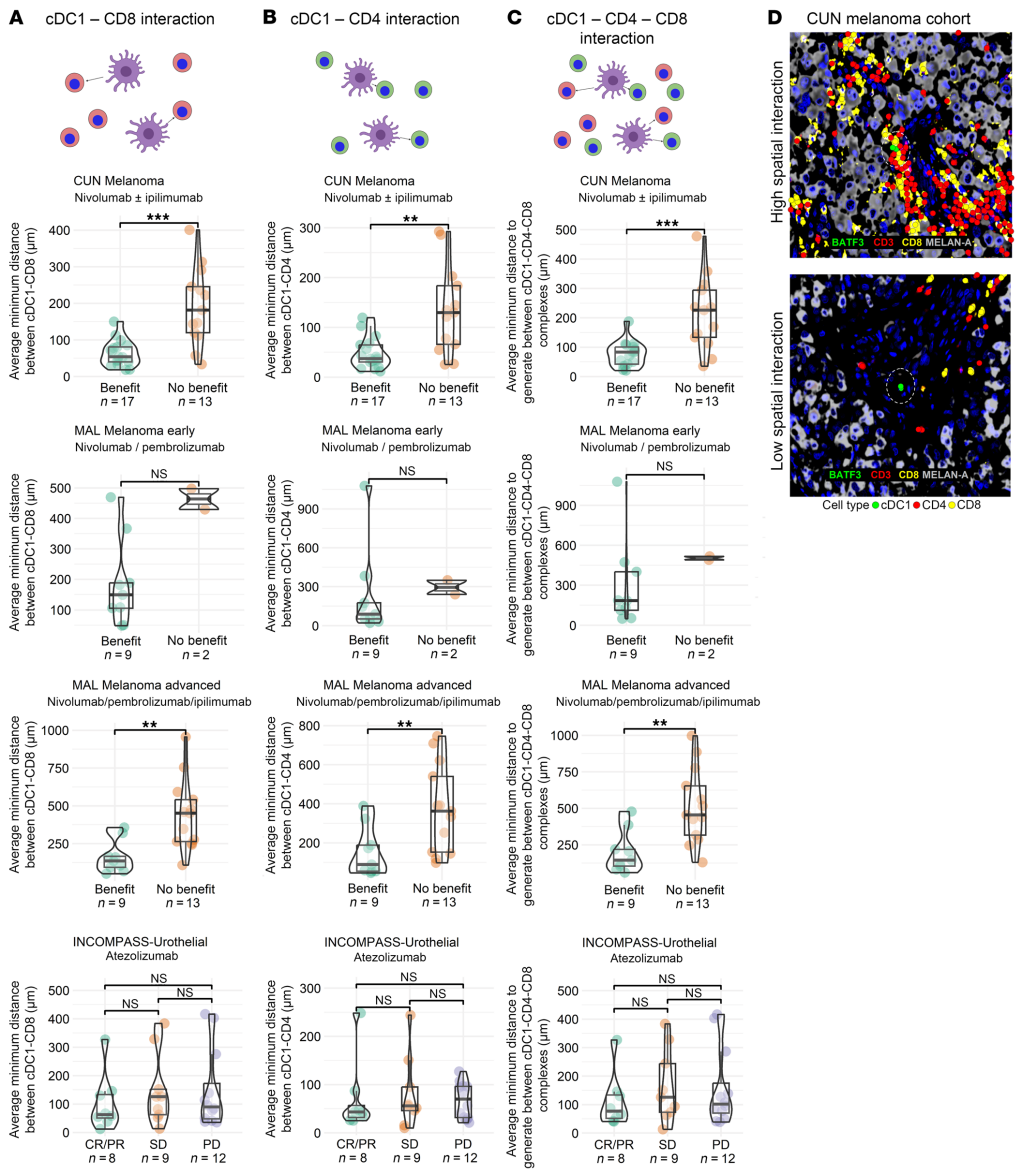
Analysis of the associations of distances between cDC1 cells and CD8^+^ or CD4^+^ T cells and clinical outcome. Series of tumor biopsies, as in Figure 4, were studied for the average minimum distance between cDC1 cells and CD8 or/and CD4^+^ T cells. **A** represents cDC-to-CD8^+^ T cell distance according to clinical outcome in the 4 series of biopsies. **B** represents cDC1-to-CD4^+^ T cell distance according to clinical outcome in the 4 series of biopsies. **C** represents compiling distances among cDC1-to-CD4-to-CD8 cells, representing triads. (**D**) Representative image analyses from 2 cases in the CUN melanoma cohort. Circles have a radius of 20 microns. Asterisks indicate *P* values for comparisons according to Wilcoxon significance tests: *P < 0.05, **P < 0.01, ***P < 0.001.

We analyzed if the spatial configuration of immune populations could further refine the predictive ability of our multiplex immunofluorescence data. To this end, we calculated the average minimal distance between cDC1 cells and T cell subsets in those samples that harbored at least a single cell from each cell type. Shorter distances between cDC1 and CD8^+^ T lymphocytes were strongly associated with clinical benefit (Figure 5A). A similar but weaker association was observed for the distance pattern between cDC1 and CD4^+^ T cells (Figure 5B). Importantly, when we analyzed the average minimum distance to form a triad between a cDC1 cell and a CD4^+^ and a CD8^+^ lymphocyte, we observed that patients benefiting from immunotherapy had shorter distances (Figure 5C). However, the strongest association pattern was observed with regard to CD8-to-cDC1 distances. These results indicate that tissue neighborhoods denoted by proximity of cDC1 and CD8^+^ T cells show the most consistent association with clinical benefit. Representative microphotographs in Figure 5D indicate that distances were variable.

### Spatial transcriptomic profiling of melanoma and NSCLC samples demonstrates CD8^+^ T cell activation in cDC1-rich neighborhoods.

**Figure 6:**
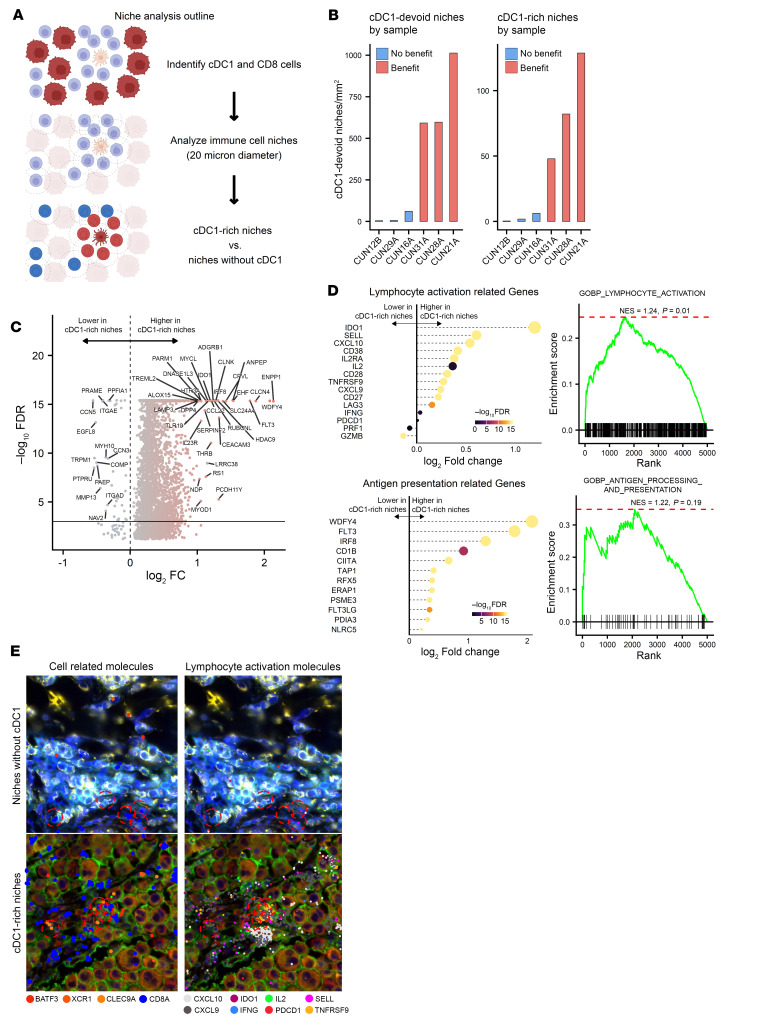
Xenium spatial transcriptomic analyses of immune related hubs showing associations with T cell functional transcripts. 6 representative cases from the CUN melanoma cohort with different densities of cDC1 cells were subjected to Xenium spatial transcriptomic analyses using the PRIME 5K panel. **A** represents the strategy to define immune related niches by the area of 20-μm diameter (10-μm radius) around an immune cell identified by their key distinguishing transcripts. Niches were classified according to the presence of cDC1 or the absence of cDC1 transcripts. (**B**) Bar plots showing the density of immune niches with or without cDC1 cells according to clinical benefit. (**C**) Volcano plot representing the transcripts enriched in immune niches containing cDC1 transcripts versus those without them. (**D**) Key hand-picked transcripts corresponding to lymphocyte activation (upper panels) and antigen presentation (lower panels), which were differentially expressed in cDC1-rich versus cDC1-devoid immune niches. Point sizes reflect fold change values. The corresponding GSEA according to gene ontology are provided. NES, Normalized Enrichment Scores. (**E**) Representative images of the immune niches used for analyses with the located transcripts represented by color-coded dots. Circles represent analyzed 20-μm diameter immune niches. On the left, images representing transcripts used to identify cell types and on the right some transcripts denoting T cell activation. The upper images represent an area with cDC1-devoid immune niches and lower images show a representative image of cDC1-enriched immune hubs.

We selected 6 cases from the CUN melanoma cohort (3 responders and 3 nonresponders, see Methods) to undergo spatial transcriptomic analysis using the Xenium Prime 5K panel. After cell segmentation, transcript counting and quality control filtering, we included 863,818 cells in the analysis. The median molecular count by cell was 230 (percentile 25–75 = 154–356). We identified 92,609 potential immune cells (either CD8 or cDC1) that would be visited by our neighborhood identification algorithm to interrogate the composition of immune niches. These immune niches/hubs consisted of 20-micron diameter areas (314 squared microns) that harbored transcripts defining either CD8, cDC1, or both cell types simultaneously. Hubs defined in this way contained between 1 and 11 cells (median 4). We compared immune hubs where cDC1 presence could be detected (9,245 cDC1 rich hubs based on BATF3, XCR1, and CLEC9A) with those in which cDC1 transcripts were absent (83,364 hubs) (Figure 6A). Figure 6B shows the relative density of cDC1-rich hubs in the samples from the 3 responding and the 3 nonresponding patients, which were consistent with the results of the multiplex immunofluorescence analyses.

Comparing immune niches with and without cDC1 cells (Figure 6C), we found that genes related to IFN-G signaling (IDO1, CXCL10, CXCL9) and T cell activation (IL2-RA, TNFRSF9/ 4-1BB) were significantly overexpressed in cDC1-rich hubs. Furthermore, transcripts denoting lesser effector differentiation such as CD62L (SELL) and CD28 were also observed. On the contrary, genes denoting terminal effector differentiation (like PRF1 and GZMB) were less expressed in cDC1-rich hubs compared with those without cDC1 (Figure 6D). These differences were further supported by the results of GSEA. Figure 6D also shows enrichment of genes denoting active antigen presentation and confirming cDC1 cell presence in the defined cDC1-rich immune hubs. Furthermore, B cell associated transcripts (such as BCL6, CD19, CD79A, CD38) and germinal center–associated pathway were found to be upregulated in cDC1-rich immune niches (Supplemental Figure 6A). In Figure 6E, representative images of immune hubs featured as containing cDC1 versus those in which cDC1 were absent are shown, detailing cell-identifying transcripts as well as some T cell activation transcripts. Our results strongly indicate a functional and spatial interplay of cDC1 and CD8^+^ T cells that is associated with the functional status of antitumor immune responses.

GeoMX DSP analysis of the YALE-NSCLC surgical cohort demonstrated results in line with the Xenium Melanoma analysis. We observed a positive correlation of BATF3 transcripts in the tumor compartment with other key transcripts associated with antigen presentation (WFDC3, CIITA, TAP1, ERAP1), cDC1 migration and survival (FLT3LG), and immune activation (CXCL9, TNFRSF9, IL2RA and IRF8) (Supplemental Figure 6B). In addition, we found a positive nonsignificant association between BATF3 mRNA presence in the tumor and patient survival (Log-Rank *P* value = 0.23, Supplemental Figure 6C). Further, we also observed an association of BATF3 transcript abundance with improved survival according to expression deciles (HR per decile increase = 0.95, *P* value = 0.22).

## Discussion

The events leading to response to immunotherapy with checkpoint inhibitors are the subject of intensive analysis in a quest for biomarkers and actionable mechanisms associated to response or resistance (32, 33). The pioneering work of Antoni Ribas’ group defined that CD8^+^ T cell density in melanoma was clearly associated with clinical outcome following PD1 blockade (25). Moreover, relatively simple gene signatures denoting CD8^+^ T cell infiltration with signs of activation have been associated with likelihood of response (26). Bulk and single-cell mRNA-seq datasets have also indicated that T cell density and functional status are associated with clinical benefit from checkpoint inhibitors or with the lack of it (34, 35).

For the priming and reinvigoration of antigen-specific CD8^+^ T cell responses, the contribution of cDC1 dendritic cells is believed to be paramount (1). On the one hand, these cells are able to present tumor antigens associated with their MHC class-I moieties and, on the other hand, provide powerful costimulation if the cDC1 have been stimulated by microbial patterns (36) or activated by CD40L^+^ CD4^+^ T helper cells (37).

Experiments in mouse models of checkpoint immunotherapy conclusively show the necessity for these cDC1 cells (12, 13) and their performance in the tumor tissue, (38) where they functionally interplay with CD8 and NK lymphocytes (14) perhaps in addition to further functional interactions that might take place in tumor-draining lymph nodes. In addition, animal experimental models have shown a spatial architecture of cDC1 cells, also conditioning their spatial interactions with CD8^+^ T cells (39, 40).

All of these pieces of information predicted that, in the tumor microenvironment of cancer patients, we ought to find a strong correlation of cDC1 density and clinical benefit from immunotherapy, as previously suggested by others in small series of melanoma and breast cancer cases (17–20). In the present work, we show that such conclusion is valid across tumor types and immunotherapy treatments, studying very well clinically annotated datasets from key industry sponsored clinical trials (41–47). Our results demonstrate the solid grounds of this predictive association for clinical outcomes that were not substantiated in the case of patients treated with TKIs or chemotherapy in the control arms of 4 of such trials that were randomized. This indicates that cDC1 abundance is a predictive rather than a prognostic biomarker, at least for objective response.

The microenvironment of solid tumors is complex and the interaction of cDC1 and CD8^+^ T cells has been postulated to be a critical factor (1). Cell-to-cell interactions can be estimated by measuring distance patterns between cells. Indeed, in 4 independent series, we have observed a clear predictive value for cDC1-to-CD8 T cell short distances, although cDC1 to CD4^+^ T cell distances also showed a certain degree of statistical significance. Interestingly, we found that the minimal distance required to generate cDC1/CD8/CD4 complexes also correlated with clinical outcome. This could be reminiscent of the triads reported to be beneficial by Miriam Merad’s group in HCC patients treated with anti-PD1 as a neoadjuvant treatment, which favorably correlated with pathological response (7). cDC1 interactions with NK cells were also spotted in our studies, although with a weaker coefficient of correlation. The interplay between cDC1 and CD4^+^ T cells was mainly interpreted in the sense of cDC1 licensing via CD40L and IFN-1 (2, 48). In the case of NK cells, M. Krummel et al. also had reported an interesting clinical association in a small series of melanoma biopsies (14). According to our analysis, the interaction of cDC1 and NK cells seems to be of lesser importance to predict clinical outcome compared with that between CD8^+^ T cells and cDC1s. Furthermore, our spatial transcriptomic analysis revealed overexpression of B cell–related transcripts in cDC1-rich immune niche, suggesting a role of these cells in the formation of tertiary lymphoid structures, as shown by recent evidence prepublished by the group of Miariam Merad (24).

A key remaining question is what the factors that bring cDC1 cells into the TME are. Our results show a strong correlation of cDC1 presence with CCL4 and CCL5 chemokine transcripts. This is reminiscent of previous publications in mouse models of melanoma and HCC (17–20). Some degree of correlation was also observed between cDC1 presence and FLT3L transcripts, as proposed by M. Krummel et al. and confirmed by our spatial transcriptomic analysis. Of note, in that report the main cells producing FLT3L were the intratumoral NK cells (14). In contrast, XCL1 was not clearly correlated, indicating that perhaps this exclusive CD8/NK–cDC1 chemokine axis is not as relevant to determine density of cDC1 cells in the malignant tissue. Nonetheless, this chemokine axis might be important to determine the distance between cDC1s and T and/or NK lymphocytes as we have and are currently exploring in mouse models and human tissue samples (31).

Spatial analysis is becoming critical to understand the heterogeneity of the tumor microenvironment (49). It characterizes the spatial contextualization of the cellular diversity of tumors. Beyond contextualizing individual cell types, we established the cellular and spatial niche context of cDC1-CD8^+^ T cell interactions in advanced melanomas. As key processes, antigen crosspresentation and crosspriming take place in a spatially coordinated manner. Therefore, characterizing these niches as distinct functional units allows direct investigations on correlates for clinical efficacy of immunotherapy.

Hubs of T cell activation have been described in tumor tissues, containing T cells undergoing activation and professional antigen presenting cells (7, 50). As an important example of spatial configuration relevance, the presence and density of B cells organized in tertiary lymphoid structures (TLS) have been shown to correlate with favorable immunotherapy outcomes across malignant diseases (51). Our analyses of hubs containing cDC1 cells indicate that this spatial interaction is functionally important to drive benefit from immunotherapy. Hubs containing cDC1 cells showed transcripts associated with immune activation and antitumor activity.

There are some limitations to our spatial transcriptomic study. First, while this is the first imaging-based spatial transcriptomic study of cCD1-CD8 interaction in melanoma and NSCLC reported to date, this study ultimately reflects a relatively small number of samples. Additionally, cell segmentation remains a challenge, particularly in melanoma, where tumor cells are negative for the typical epithelial markers used in the Xenium immunofluorescence workflow. To circumvent this problem, we resourced to study niches of 20 micrometers in diameter rather than single cells. Validation of these data in a larger series of NSCLC samples studied by GeoMX was performed, concluding similar associations. A parameter that becomes important is tumor tissue heterogeneity, as previously defined for Tumor Infiltrating Lymphocyte density (52). Indeed, our ongoing research is addressing those aspects for cDC1.

All considered, our data conclude beyond doubt that cDC1 density and function and their interaction with CD8^+^ T cells in the tumor microenvironment are key factors for immunotherapy efficacy and hold promise as a tumor type–agnostic biomarker. These facts also pinpoint to a network of mechanisms that can be defective in those patients who do not respond to immunotherapy. Moreover, these results suggest potentially actionable routes for combined strategies of treatment (53, 54) by means of acting on those defective mechanisms that ought to be mediated by cDC1 cells.

## Methods

### Sex as a biological variable.

Both sexes were included in the present study. Sex was not considered as a biological variable during data analysis.

### Transcriptomic datasets.

A detailed description of the transcriptomic datasets analyzed can be found in the Supplemental Methods. In brief, we retrieved RNA-seq data from 7 pivotal trials of immune checkpoint inhibitors. The IMmotion150 trial (ClinicalTrials.gov: NCT01984242) included patients diagnosed with metastatic renal cell carcinoma (mRCC) who had not undergone prior systemic therapy (41, 42) to receive atezolizumab, atezolizumab + bevacizumab or Sunitinib. The IMbrave150 trial (ClinicalTrials.gov: NCT03434379) evaluated the efficacy of atezolizumab (anti-PD-L1) in combination with bevacizumab (anti-VEGF) versus sorafenib as first-line therapy for patients with unresectable hepatocellular carcinoma (HCC) (43). The POPLAR study (ClinicalTrials.gov: NCT01903993) evaluated the efficacy of atezolizumab versus docetaxel in patients with previously treated non-small-cell lung cancer (NSCLC) (44). The OAK trial (ClinicalTrials.gov: NCT02008227) evaluated the efficacy of atezolizumab compared to docetaxel in patients with previously treated, locally advanced, or metastatic NSCLC with platinum-based chemotherapy (45). Gide et al. (46) investigated immune responses in patients with metastatic melanoma, comparing anti-PD-1 monotherapy (e.g., pembrolizumab or nivolumab) and combination therapy with anti-PD-1 and anti-CTLA-4 (ipilimumab). Riaz et al. (55) studied patients with metastatic melanoma treated with nivolumab±ipilimumab. The IMvigor210 (47) (ClinicalTrials.gov: NCT02108652) trial evaluated the efficacy and safety of atezolizumab in patients with locally advanced or metastatic urothelial carcinoma (mUC).

### RNA-seq sample preprocessing and analyses.

All sample processing and subsequent bioinformatics analyses were performed on a workstation equipped with 16-core Intel Xeon W-2245 @ 4.7 GHz and 256 GB of RAM, running Linux (Ubuntu 20.04). For repositories with restricted access, raw data files and clinical data deposited in EGA (56) were retrieved following the approval of access permissions. The same analytical pipeline was subsequently applied to the cohorts available in the GEO repository (57), except for the Gide et al. dataset, for which only normalized counts were available (log2-TPM). Only pre-treatment patients were included in the analysis. The initial quality control of the samples was performed using the FastQC tool (v0.11.9) (58). Low-quality reads and adapter sequences were subsequently removed with Trimmomatic (v0.39) (59) for paired-end data (e.g., ILLUMINACLIP:(adapters.fa):2:30:10 SLIDINGWINDOW:(4:20) MINLEN:30). Alignment was performed using STAR(v2.7.9a) (60) with the hg38 assembly as the reference genome. Raw counts were obtained with featureCounts (v.2.0.0) (61) and annotated with Gencode gtf (v38) (62). Counts normalization and subsequent analyses were conducted using R/Bioconductor statistical environment (v4.1.1). Genes with zero counts across all samples were removed prior to normalization. For each dataset, input expression values were log2-CPM computed with effective library sizes (edgeR TMM normalization, normalized.lib.sizes=TRUE), and transformed to log2 counts per million (log2-CPM) to enable within-gene, between-sample comparisons. For the Gide et al. cohort, analyses were performed on the provided (log2-TPM)values using settings consistent with log-normalized input.

### cDC1, NK and CD8 signature construction.

To derive robust and broadly applicable signatures, we started from the MSigDB C8 collection (cell-type gene sets) and retrieved all lists annotated to dendritic cells (DC), CD8^+^ T cells, and NK cells. Within each cell type, the available lists were merged (union) to form an initial candidate pool. We then applied a cohort-wise detectability filter to prioritize broadly expressed genes: a gene was retained if it showed ≥ 5 raw counts in ≥ 50% of patients within a cohort (for the Gide cohort, ≥ 1 TPM in ≥ 50% of patients).

Next, for each cohort and cell type, we computed a per-sample meta-signal from the retained candidates and quantified each gene’s importance via its Spearman correlation with that meta-signal. Genes with R>0.4 and significant p-values were kept, and we focused on the intersection across cohorts to ensure cross-study robustness. Finally, we refined the lists into cell type–specific signatures (cDC1, CD8, NK) through an exhaustive literature review of canonical markers and functional evidence (15, 63–70).

### Signature score computation and correlations.

Signature score was performed in R (v(4.1.2))/Bioconductor using the GSVA package (v2.0) (71), with method=”gsva” and the package′s default parametrization for continuous, log2-normalized inputs (i.e, Gaussian kernel). No additional tuning of GSVA parameters were applied. To facilitate interpretation and visualization across figures, GSVA scores were min–max rescaled to a 1–10 range within each cohort/platform. Min-max scaling was computed as:

Equation 1


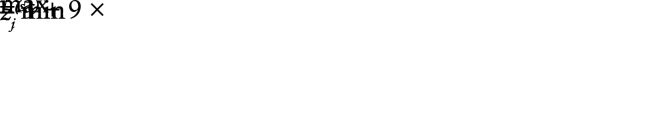


where
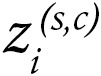
 is the original GSVA score for sample *i*, signature *s*, in cohort *c*; the minimum and maximum are computed intracohort (and, if applicable, per signature). The scaled score
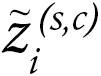
 values lie in in between 1 and 10. For single-gene summaries we used log_2_(CPM + 1) (or log_2_(TPM + 1) when TPM was the original unit) to avoid negative values and stabilize variance. Unless otherwise stated, statistical comparisons (correlations, between-group tests) were performed on the original expression or signature score prior to any rescaling, and the log transform with a +1 pseudocount was applied for interpretability and visualization only.

All statistical tests were conducted on the unscaled GSVA values. Patients were stratified by clinical response (PR, CR, SD, PD); due to the small number of CR cases, PR and CR were grouped. Group comparisons used the Mann–Whitney U test (two-sided). Pearson correlations among signatures or between signatures and transcripts were computed in base R, and visualizations were produced with ggplot2 (v3.5.1) (72) and ggstatsplot (v0.12.4) (73).

Regarding missing value handling, all signature genes passed the detectability filter across cohorts except NCR1 and KIR2DL1, which fell below the threshold in some cohorts. We retained these genes to preserve the biological completeness of the NK signature. We did not impute undetected values. GSVA was run on the available genes in each cohort’s expression matrix (genes absent from a cohort’s feature space are automatically ignored).

All analyses were performed within each clinical trial/platform. No cross-cohort batch correction (e.g., ComBat) was applied because the primary sources of batch were tissue-of-origin and sequencing platform.

### Multiplex immunofluorescence.

TMAs or whole slide samples were pre-processed following previously reported workflows (74). The multiplex immunofluorescence panel was developed as previously described by our group (75). A panel targeting BATF3, CD3, and CD8 was built. Samples were stained using a Leica BOND RX autostainer. Samples were de-paraffinized and rehydrated before heat-induced antigen retrieval. Antigen retrieval was performed at high pH conditions (pH 9, target retrieval solution Dako-Agilent catalogue # S2367). Primary antibodies included CD8 (Pre-diluted, clone C8/144B, DAKO, catalogue #M710301), CD3 (Pre-diluted, Polyclonal, DAKO, catalogue #GA50361-2), and BATF3 (1:500, Polyclonal, R&D SYSTEMS, catalogue #AF7437). Additionally, for the melanoma samples we also tested MELAN-A protein (1:200, clone A103, Dako, catalogue #M7196). Before each round, antigen block was performed with 20% goat serum albumin diluted in PBS (catalogue #X0907, Dako-Agilent). Protein detection was performed using biotinylated secondary antibodies and streptavidin-HRP complexes (catalogue #P0397, Dako-Agilent). Afterwards, tyramide signal amplification (TSA) was performed using Opal fluorophores (570, 520, 620, and 480, catalogue numbers #FP1488001KT FP1487001KT, FP1495001KT and FP1500001KT, respectively) diluted in 1X Plus Amplification Diluent (catalogue #FP1609, Akoya Biosciences). The final round included nuclear staining using spectral DAPI (catalogue #FP1490, Akoya Biosciences). Afterwards, the coverslip was placed using Faramount Aqueous Mounting Medium (catalogue #S3025, Dako-Agilent).

### Multiplex immunofluorescence tumor tissue samples.

Four cohorts of patients were included in the study (Supplemental Table 1). The first cohort included 34 patients diagnosed with advanced, non-operable urothelial carcinoma enrolled in the registered clinical trial (NCT Eudract) (76) (INCOMPASS Cohort). All patients were treated with Atezolizumab (administered every 3 weeks until progression or intolerance). Patients were followed until death from disease. Progression from disease was also annotated. In addition, radiologic response to systemic treatment was studied according to clinical trial protocol and reported according to current RECIST criteria (version 1.1). Formalin Fixed Paraffin Embedded (FFPE) tissue samples from surgical resections were reviewed by an experienced pathologist. From each FFPE tissue block up to two 1mm cores were obtained from representative tumor areas to build a Tissue Micro Array (TMA) containing 47 evaluable tissue cores (21 patients provided 1 core, 13 patients provided 2 cores). Of the evaluable patients, ten experienced a complete response or partial response (24.4%) and ten had a stable disease (24.4%). The remaining 14 patients experienced tumor progression (36.6%) as their best response. Median overall survival was 9.09 months and median PFS was 5.22 months (p25-p75: 2.08 – 9.36). Before being included in the trial, patients had received between 1 to 4 previous systemic treatment lines.

The second cohort included 30 patients diagnosed with advanced melanoma (CUN Melanoma). Patients were treated with systemic immunotherapy based on Nivolumab or a combination of Nivolumab+Ipilimumab. Benefit from treatment was measured at 12 months by RECIST criteria as above. For each patient, a FFPE sample from the initial tumor resection was obtained. Whole slides from the FFPE tissue block were used for immunofluorescence analysis. At 12 months from treatment initiation, 17 patients experienced a shrinkage of their metastasis (54.8%), while there was not an objective benefit in the remaining 13 patients (45.2%).

The third and fourth cohorts included patients diagnosed with metastatic melanoma (MAL Melanoma advanced *n* = 20) and early-stage melanomas respectively (MAL Melanoma early *n* = 11). Metastatic melanomas were treated in their majority with Nivolumab in monotherapy, with 4 patients treated anti-PD1+TKI and a single patient receiving anti-CTLA4 combinations. Early-stage melanomas received adjuvant anti-PD1 treatment (Pembrolizumab or Nivolumab) after surgery. Metastatic patients were categorized as good or bad responders as previously published (77). In brief, patients were categorized as bad responders if progression occurred in less than 3 months after treatment initiation. Good responders were defined as patients demonstrating partial or complete response for a year or that stayed in treatment for at least 12 months. For early-stage patients receiving adjuvant checkpoint blockade, good responders were those not presenting relapse/metastasis in the first 12 months after start of treatment, and bad responders those that progressed within the first 3 months.

In both cases, the closest FFPE biopsy to the start of the immunotherapy treatment was selected. Two patients from the MAL Melanoma advanced cohort provided two tissue samples. From each sample a whole slide obtained from the FFPE tissue block was used for further analyses. Of the 22 samples analyzed from 20 evaluable patients included in the metastatic setting, 9 were good responders, 13 bad responders.

Of the 11 patients who were recruited in the adjuvant setting, 9 did not have relapse/metastasis during the first 12 months after treatment started, and 2 patients experienced progression in the first 3 months after initiation of adjuvant immunotherapy.

As a validation in a cohort of samples from another clinical center, we included a cohort of patients diagnosed with non-small cell lung cancer and treated with immunotherapy. Formalin-fixed paraffin-embedded (FFPE) tumor tissue samples from a retrospective cohort of NSCLC patients treated with immunotherapy at the Yale-New Haven Hospital from 2011–2020 which were analyzed in tissue microarray (TMA) format (78–80). TMAs were built by combining several individual 0.6-mm (diameter) cores from each tumor sample with each region of interest selected by a trained pathologist to be representative of the tumor lesion. All tissue and clinical information were used after approval from the Yale Human Investigation Committee protocols #9505008219 and #1608018220 or local institutional protocols, which had approved the patient consent forms or a waiver of consent.

### Tissue imaging and analysis.

Multiplexed immunofluorescence TMA slides were scanned on a PhenoImager HT Automated Quantitative Pathology Imaging System (Akoya Biosciences). Briefly, a spectral library containing the spectral peaks emitted by each fluorophore from single stained slides was created using inForm software (version 2.4.8, Akoya Biosciences). This spectral library was used for spectral unmixing of the images, allowing color-based identification of the markers of interest. Autofluorescence was determined on an unstained urothelial carcinoma tissue. Each tissue sample image was spectrally unmixed and exported as a component TIF image using Akoya Biosciences’ Inform software. Component TIF images were then imported into the open-source digital pathology software QuPath version 0.2.3 (University of Edinburgh, Edinburgh, UK; https://qupath.github.io).

Cell segmentation was performed based on the DAPI channel (for nuclear segmentation) and the CD3 and CD8 channel (for membrane segmentation) using the Mesmer segmentation pipeline through plugins made for QuPath (81). Cells close to the border of the images were removed to reduce the risk of staining artifacts or phenotyping errors. Afterwards, cell segmentation masks were used to obtain every cell centroid and the average pixel intensity for every cell and channel. As a result, a cell feature matrix containing the X and Y cell coordinates and the average marker intensity for every cell in each image were obtained. This matrix was used to assign a single phenotype label to each cell. To this end, marker intensity values were thresholded using the Triclass Otsu method implemented in the imagerExtra R package (ver 1.3.2) using the CSM r package (82, 83). Marker positivity patterns were used to assign a cell-type label. Cells were labelled as cDC1 if they were positive for BATF3. BATF3 negative cells were further classified as cytotoxic T lymphocytes if they were CD8 positive. T helper (CD4 positive) lymphocytes were identified as cells demonstrating CD3 positivity and that were CD8 negative. Cells that were negative for all the markers (BATF3, CD8 and CD3) were labelled as “other”. Tissue size was estimated using the cell coordinate location from the cell feature matrix using the Image_size_calculator CSM function. Afterwards, cell densities were calculated. To calculate densities, the whole tissue area was considered, including tumor and stromal areas. Then, spatial interaction analyses were performed. Samples devoid of BATF3 positive dendritic cells or lymphocytes were excluded from the analysis. To find interaction patterns, the average distance between BATF3 positive dendritic cells and both CD4 and CD8 lymphocytes was calculated using custom R scripts. In addition, the minimum distance from every BATF3 cell to interact simultaneously with a CD4 and a CD8 lymphocyte was calculated (defined as the minimum distance required for every BATF3+ cDC1 cell to encounter a CD4 and a CD8 lymphocyte).

For the NSCLC samples, we used an alternative approach to quantify the density cDC1 cells. In brief, a multiplexed quantitative immunofluorescence (QIF) panel was established to detect cDC1s by the coexpression of the cDC1 markers XCR1, CD11c, and HLA-DR, including pan-cytokeratin (CK) to mark tumor epithelial cells and DAPI to stain the nuclei (84). Briefly, TMA sections were sequentially deparaffinized, rehydrated, and treated with antigen retrieval buffer (1 mM EDTA, pH 8.0) in a pressure-boiling module (Lab Vision) at 97ºC for 20 min. Slides were further incubated in methanol containing 0.75% hydrogen peroxide at RT for 30 min and later in blocking solution (0.35% bovine serum albumin and 0.05% Tween-20 in 1X TBS) at RT for 30 min. Sections were treated overnight at 4°C with a primary antibody solution containing rabbit monoclonal anti-CD11c IgG (Cell Signaling Technology, D3VE1 clone, cat. No. 45581) and mouse monoclonal anti-HLA-DR IgG2b (LifeSpan Bio, 4C8 clone, cat. No. LS-C133245), both diluted at 1:200.

The next day, slides were sequentially incubated with secondary antibodies for 1 h at RT followed by fluorophore-conjugated tyramide molecules for 10 min at RT: anti-Rabbit PowerVision Poly-HRP (Leica, cat. No. PV6119) & TSA Cyanine 5 reagent (Akoya Biosciences, SAT705A001EA) for CD11c, and Goat anti-mouse IgG2b HRP-conjugated polyclonal (Abcam, cat. No. ab97250) & TSA Plus Cyanine 3 (Akoya Biosciences, cat. No. NEL744001KT) for HLA-DR. Residual HRP was quenched in-between secondary antibody-tyramide incubations by treating the sections twice for 7 min with 1X PBS containing benzahydrazide (Sigma) and 50 μl of 30% (w/w) hydrogen peroxide. Rabbit monoclonal anti-XCR1 IgG (Cell Signaling. Technology, D2F8T clone, cat. No. 44665) was then added for 1 h at RT, followed by Goat anti-rabbit IgG HRP-conjugated polyclonal (Abcam, cat. No. ab6721), also for 1 h at RT. Finally, TSA Biotin reagent (Akoya Biosciences, SAT700001EA) was added for 10 min at RT, followed by co-incubation of Alexa Fluor 750-Streptavidin (Thermo Fisher, S21384) and Mouse monoclonal anti-pan-CK IgG1, Alexa Fluor 488-conjugated (Thermo Fisher, AE1/AE3 clone, cat. No. 53-9003-82) for 1 h at RT. Nuclei were counterstained with 4’,6-diamidino-2-phenylindole (DAPI). Washes with 1X TBS and 1X TBST (TBS with 0.1% Tween 20) were done for 1 min after application of each reagent. Slides were scanned at 20X magnification using the PhenoImager HT 2.0 Instrument (Akoya Biosciences). A single-representative tissue core for every of the 130 patients included in the present study was analyzed. Individual cell phenotyping for cDC1s calculated as cell density (number of cells/tissue area in mm^2^) was determined by the co-localization of markers XCR1, CD11c, and HLA-DR in tissue using the inForm software v.2.6 (Akoya Biosciences). In order to analyze association between cCD1 cell density and survival, we first classified samples as low or high cDC1 content using the median cDC1 expression as cuT-off point. Afterward, Log-Rank analysis and Kaplan-Meier plots were analyzed to find association with overall survival. In addition, we computed a Cox Proportional Hazard model using cDC1 density deciles as independent variable.

### Xenium Prime 5K panel spatial transcriptomics.

In order to analyze the transcriptomic impact of cDC1 on CD8 lymphocytes, we analyzed 6 melanoma samples from the advanced melanoma cohort by Xenium spatial transcriptomics (10x Genomics). To this end, 3 samples that showed high CD8-cDC1 spatial interaction and experienced a benefit from immunotherapy and 3 additional samples demonstrating low spatial interaction and experiencing adverse clinical outcomes were used. First, immunofluorescence images from these samples were reviewed, and a single 5x5mm ROI containing cDC1 and CD8^+^ T cells was selected per sample. Tissue blocks were macroscopically dissected to isolate this 5x5mm tissue fragments. Afterwards, tissue slices were analyzed using the Xenium Prime 5K gene expression panel.

Data generated from the analysis was first pre-processed using the Seurat R package (ver5.2.1) (85). Cell feature matrix were first filtered to remove cells with less than 50 total molecules identified. In order to analyze impact of spatial interaction on cell transcriptome, we performed niche analysis similar to previously published pipelines (86). To this end, we first spotted potential CD8 or cDC1 cells. We used 2 combined approaches to annotate these potential cDC1 and CD8^+^ T cells in the data. The first method was threshold based, selecting cells whose sum of expression for a list of canonical genes (BATF3, CLEC9A and XCR1 for cDC1 and CD8A and CD8B for cytotoxic lymphocytes) is above the local minimum calculated in the bimodal distribution that characterizes the sum of expression for those genes. The second method was based on a finely granulated unsupervised clustering (Leiden algorithm). Clusters were subsequently merged based on expression of the canonical genes for one or the other cell type, both (annotated as “mixture”) and for those having none. The annotation of both methods for each cell was crossed in order to reach a common consensus, checking against some canonical genes in order to minimize noise input by surrounding malignant cells. Afterwards, these potential immune cells were subjected to message passing, a process by which neighboring cells share their transcriptomes. To this end we used the UTAG algorithm (86) adaptation implemented in the CSM R package (83). We selected a 10-micron distance to define neighboring cells (Niches). After performing message passing, niches contained the transcriptomic information of cells whose centroids are within a 10-micron distance from the niche centroid. In order to analyze niches enriched in immune cells, the algorithm only visited cells that were identified as potential CD8 or cDC1 cells as described above.

We analyzed the transcriptomic differences between immune niches that showed cDC1 presence and those with absence of cDC1 cells. To that end, we first classified niches as cDC1-rich if they showed at least the presence of a single cDC1-related molecule (either BATF3, XCR1, or CLEC9A). Those without cDC1 molecules were considered to be cDC1 devoid. To calculate differential expression, we used the Zero-Inflated Negative Binomial model implemented in the Bioconductor R package DEsingle (ver 1.26.0) (87). Afterwards, Gene Set Enrichment analysis was conducted using the fgsea Bioconductor R package (ver 1.32.4) (88). Gene ontology pathways were obtained through the msigdbr package (ver 10.0.1) (89).

### GeoMX spatial transcriptomics analysis.

In order to perform spatial transcriptomics analysis of the Yale-NSCLC cohort, a subset of the TMA samples used in the mIF series was selected to undergo Nanostring GeoMX Digital Spatial Profiler analysis. To this end, a set of 110 tumor cores were analyzed. Transcriptomic information from the pan-cytokeratin positive compartment (tumor compartment) was used for further analyses. Before proceeding with analysis, GeoMX expression data was first subjected to quality control checks and samples with less than 50,000 raw counts were removed. Count matrix size factor normalization was performed using the geomxNorm function implemented in the standR Bioconductor package (1.6.0) (90). Normalized BATF3 transcript counts were used as a surrogate of cDC1 presence and abundance in the tumor compartment. In order to find associations with overall survival, we used the median BATF3 expression as a cut-off point as described above for mIF experiments. Afterwards, Log-rank test analysis was conducted. Further, we computed a Cox Proportional Hazard model by dividing samples according to their BATF3 transcript expression deciles. In addition, we also calculated the Spearman correlation rho between BATF3 mRNA expression level and various key immune mediators identified in our previous Xenium spatial transcriptomic analysis.

### Statistics.

Between-group comparisons were performed using the Wilcoxon test. Correlation between transcriptomic signatures was measured using the Pearson correlation index. Correlation between multiplex immunofluorescence cell densities was measured using Spearman correlation index. Correlation between gene transcript expression in spatial transcriptomic data was analyzed using Spearman correlation index. Statistical significance was reached if *P* values were below 0.05.

### Study approval.

The present work has been prepared in accordance with the declaration of Helsinki.

### Data availability.

Data used in the present work is available at reasonable requests to the corresponding author. Values for all data points in graphs are reported in the Supporting Data Values file. RNA-seq datasets were obtained with permission from the European Genome-Phenome archive (IMvigor210 and Immotion150: EGAS00001004386, Imbrave150: EGAS00001005503, OAK and POPLAR: EGAS00001005013). In addition, the Gide *et al* TMP data was obtained from the COMPASS repository (https://www.immuno-compass.com). The fastq files from the Ríaz et al. cohort were accessed from the Gene Expression Omnibus (GSE91061).

## Author contributions

IM and CEA conceived the article work. ALJ, JGG, FI, JH, AP, and RA performed experiments. ALJ, JGG, AT, CLR, DRG, JLPG, EPR, IB, SMA, MFS, IO, MRR, IR, SAA, DRG, TA, and KAS analyzed data. ALJ, JGG, CEA, and IM wrote the manuscript. ALJ was listed first as co-first author for key involvement in all the parts of the study and its coordination.

## Conflict of interest

The authors have declared that no conflict of interest exists.

## Funding support

- This project was funded by the European Union. Views and opinions expressed are, however, those of the author(s) only and do not necessarily reflect those of the European Union or the European Research Council Executive Agency. Neither the European Union nor the granting authority can be held responsible for them.
- ERC grant (RIPECROP, 101142365).
- “la Caixa” Foundation under the project code [LCF/PR/HR21/00083].
- Understanding and exploiting crosspriming in immunotherapy of cancer CrossIT funded by ref BBASELGAFERO2022-01.
- Scientific Foundation of the Spanish Association Against Cancer (TRNSC213881MELE).
- Instituto de Salud Carlos III (AC22/00026).
- European Union – NextGenerationEU funds under the Plan de Recuperación, Transformación y Resiliencia (PRTR).
- Instituto de Salud Carlos III (ISCIII) through the project PMP22/00054 “Exploring the Feasibility of predictive and pharmacodynamics biomarkers of immunotherapy in solid tumors (Immune4ALL)” and co-founded by the European Union.
- The Spanish ministry of science innovation and university (PID2023-147515OB-I00).
- The FEDER foundation and European Union through the grant MICIU/AEI/10.13039/501100011033.

## Supplementary Material

Supplemental data

Supporting data values

## Version 1 05/01/2026

Electronic publication
