# Supplementary material for: Conventional type-1 DC density is associated with checkpoint inhibitor response across multiple types of cancer
Source: J Clin Invest. 2026 May 1;136(9):e200987. doi: 10.1172/JCI200987 (PMC13132367; doi:10.1172/JCI200987)
Supplement: Supplemental data [file jci-136-200987-s302.pdf]

Supplementary files

Supplementary methods

RNAseq datasets

Clinical characteristics of the IMmotion150 cohort

The IMmotion150 trial (ClinicalTrials.gov: NCT01984242) was a Phase II study targeting patients diagnosed with metastatic renal cell carcinoma (mRCC) who had not undergone prior systemic therapy (1,2). Participants were divided into three groups: 77 patients received atezolizumab (anti-PD-L1) alone, 85 were treated with a combination of atezolizumab and bevacizumab (anti-VEGF), while 85 patients were treated with sunitinib. In our analysis, we have combined the two groups treated with immunotherapy. Among the 162 patients treated with immunotherapy, six achieved a complete response (CR, 3.60%), 40 had a partial response (PR, 24.69%), 62 experienced stable disease (SD, 38.27%), and 54 showed disease progression (PD, 33.33%).

The median progression-free survival (PFS) was 8.10 months in the immunotherapy group, with an interquartile range (IQR) of 18.69 - 2.79 months. Among the patients treated with sunitinib, 2 achieved CR (2.35%), 26 PR (30.58%), 36 SD (42.35%) and 21 PD (24.70%). Median PFS in the sunitinib arm was 7.13 months with IQR= 16.59 - 4.37.

Clinical characteristics of the IMbrave150 cohort

The IMbrave150 trial (ClinicalTrials.gov: NCT03434379) was a pivotal Phase III study evaluating the efficacy of atezolizumab (anti-PD-L1) in combination with bevacizumab (anti-VEGF) versus sorafenib as first-line therapy for patients with unresectable hepatocellular carcinoma (HCC) (3). Most patients were in stage B or C, reflecting advanced disease. Of the total 314 patients in the immunotherapy arm, we have selected those who had a confirmed status of response (290), with RNA sequencing performed pre-treatment. Of these, 25 patients reached a complete response (8.62%), 65 partial response (22.41%), 123 stable disease (42.41%) and 77 progression disease (26.55%). Median OS in

the immunotherapy arm was 9.15 months (OS), with an interquartile range of 6.51 to 12.07. Median PFS in the immunotherapy arm was 5.55 months (p25- p75: 2.03 - 9.50). From the total number of patients treated with sorafenib, we have selected 48 with response to treatment available. None of patients reached CR, 10 patients with PR (20.83%), 24 with SD (50%) and 14 with PD (29.16%). Median OS in the sorafenib arm was 8.46 months (p25-p 75: 5.64 - 9.82) and median PFS was 4.23 months (p25-p75: 1.55 - 7.55).

Clinical characteristics of the POPLAR cohort

This Phase II study (ClinicalTrials.gov: NCT01903993) evaluated the efficacy of Atezolizumab versus docetaxel in patients with previously treated non-small-cell lung cancer (NSCLC) (4). Before immunotherapy, most participants underwent platinum-based chemotherapy, as it is a standard first-line treatment for NSCLC. We have selected those patients with confirmed response status. In the immunotherapy arm (87 patients selected), one reached complete response (1.11%), 12 partial response (13.79%), 40 patients with stable disease (45.97%) and 34 with progression (39.08%). Median overall survival was 11.10 months (p25-p75: 5.48 - 25.63). Median progression-free survival was 2.80 (p25-p75: 1.40 - 8.53). Among patients treated with docetaxel (86 selected with confirmed status response), none of them achieved CR (0%), 16 PR (18.60%), 35 SD (40.69%) and 35 PD (40.69%). Median OS in docetaxel arm was 9.38 with IQR = 16.07 - 4.21 and median PFS of 3.36 months with IQR = 6.75 - 1.38.

Clinical characteristics of the OAK cohort

The OAK trial (ClinicalTrials.gov: NCT02008227) was a Phase III study evaluating the efficacy of atezolizumab compared to docetaxel in patients with previously treated, locally advanced, or metastatic NSCLC with platinum-based chemotherapy (5). From 344 patients of the atezolizumab arm, we selected patients with confirmed response status (318) and classified them based on their response to treatment: four achieved CR (1.25%), 44 PR (13.83%), 111 SD (34.98%) and 159 PD (50%). Median overall survival was 10.77 months in

the atezolizumab arm (p25-p75: 5.48 - 25.63) and median progression-free survival was 2.80 (p25-p75: 1.40 - 8.53). From the docetaxel arm, 315 patients were annotated with confirmed response to treatment and classified by their response: None with CR (0%), 42 with PR (13.33%), 158 SD (50.15%) and 115 with PD (36.50%). Median OS in docetaxel arm was 9.10 months (p25-p75: 4.59 - 19.25) and median PFS of 3.25 months (p25-p75: 1.57 - 5.70).

Clinical characteristics of the Gide et.al cohort

Gide et al. (6) investigated immune responses in patients with metastatic melanoma, comparing anti-PD-1 monotherapy (e.g., pembrolizumab or nivolumab) and combination therapy with anti-PD-1 and anti-CTLA-4 (ipilimumab). Out of 120 patients, 63 received monotherapy and 57 combination therapy. This analysis focused on 72 patients whose sequencing before treatment. Among them, 13 achieved CR (18.05%), 26 PR (36.11%), 11 SD (15.27%), and 22 PD (30.55%). Median overall survival was 20.53 months (IQR: 29.57 - 8.35), with a median PFS of 12.4 months (IQR: 24.03 - 2.73).

Clinical characteristics of the R´ıaz et.al cohort

Riaz et al. (7) conducted a study focusing in patients with metastatic melanoma treated with immune checkpoint blockade therapy. Specifically, the study included patients who had either progressed on ipilimumab (anti-CTLA-4) or were ipilimumab-naive, and all patients subsequently received nivolumab (anti-PD-1) as part of the CA209-038 clinical trial. The research aimed to understand the evolution of both the tumor and its microenvironment during treatment with anti-PD1 therapy. In this analysis, we focused only on patients whose transcriptomic sequencing was performed before treatment and with confirmed annotated response status (49 patients). Of these, 10 patients achieved PRCR response (20.40%), 16 SD (32.65%) and 23 PD (46.93%). Median OS was 17.28 months with IQR = 29.83 - 7.85.

Clinical characteristics of the IMvigor210 cohort

The IMvigor210 (8) (ClinicalTrials.gov: NCT02108652) was a multicenter, single-arm, phase II clinical trial designed to evaluate the efficacy and safety of atezolizumab in patients with locally advanced or metastatic urothelial carcinoma (mUC). The trial comprised two distinct cohorts: The first one included patients who were cisplatin ineligible and had not received prior treatment for mUC. The second cohort consisted of patients with mUC who had experienced disease progression following platinum-based chemotherapy. We will not distinguish between the two cohorts in this analysis. A total of 208 patients underwent RNA sequencing prior to treatment. Patients OS was 9.24 months (p25 - p75: 4.12 - 20.07). Median PFS was 2.10 months (p25 - p75: 2.00 - 6.34).

References

1. Powles T, Atkins MB, Escudier B, Motzer RJ, Rini BI, Fong L, et al. Efficacy and Safety of Atezolizumab Plus Bevacizumab Following Disease Progression on Atezolizumab or Sunitinib Monotherapy in Patients with Metastatic Renal Cell Carcinoma in IMmotion150: A Randomized Phase 2 Clinical Trial. Eur Urol. 2021;79:665–73.

2. McDermott DF, Huseni MA, Atkins MB, Motzer RJ, Rini BI, Escudier B, et al. Clinical activity and molecular correlates of response to atezolizumab alone or in combination with bevacizumab versus sunitinib in renal cell carcinoma. Nat Med. 2018;24:749–57.

3. Finn RS, Qin S, Ikeda M, Galle PR, Ducreux M, Kim T-Y, et al. Atezolizumab plus Bevacizumab in Unresectable Hepatocellular Carcinoma. N Engl J Med. 2020;382:1894–905.

4. Fehrenbacher L, Spira A, Ballinger M, Kowanetz M, Vansteenkiste J, Mazieres J, et al. Atezolizumab versus docetaxel for patients with previously treated non-small-cell lung cancer (POPLAR): a multicentre, open-label, phase 2 randomised controlled trial. The Lancet. 2016;387:1837–46.

5. Rittmeyer A, Barlesi F, Waterkamp D, Park K, Ciardiello F, Von Pawel J, et al. Atezolizumab versus docetaxel in patients with previously treated non-small-cell lung cancer (OAK): a phase 3, open-label, multicentre randomised controlled trial. The Lancet. 2017;389:255–65.

6. Gide TN, Quek C, Menzies AM, Tasker AT, Shang P, Holst J, et al. Distinct Immune Cell Populations Define Response to Anti-PD-1 Monotherapy and Anti-PD-1/Anti-CTLA-4 Combined Therapy. Cancer Cell. 2019;35:238-255.e6.

7. Riaz N, Havel JJ, Makarov V, Desrichard A, Urba WJ, Sims JS, et al. Tumor and Microenvironment Evolution during Immunotherapy with Nivolumab. Cell. 2017;171:934-949.e16.

8. Rosenberg JE, Hoffman-Censits J, Powles T, Van Der Heijden MS, Balar AV, Necchi A, et al. Atezolizumab in patients with locally advanced and metastatic urothelial carcinoma who have progressed following treatment with platinum-based chemotherapy: a single-arm, multicentre, phase 2 trial. The Lancet. 2016;387:1909–20.

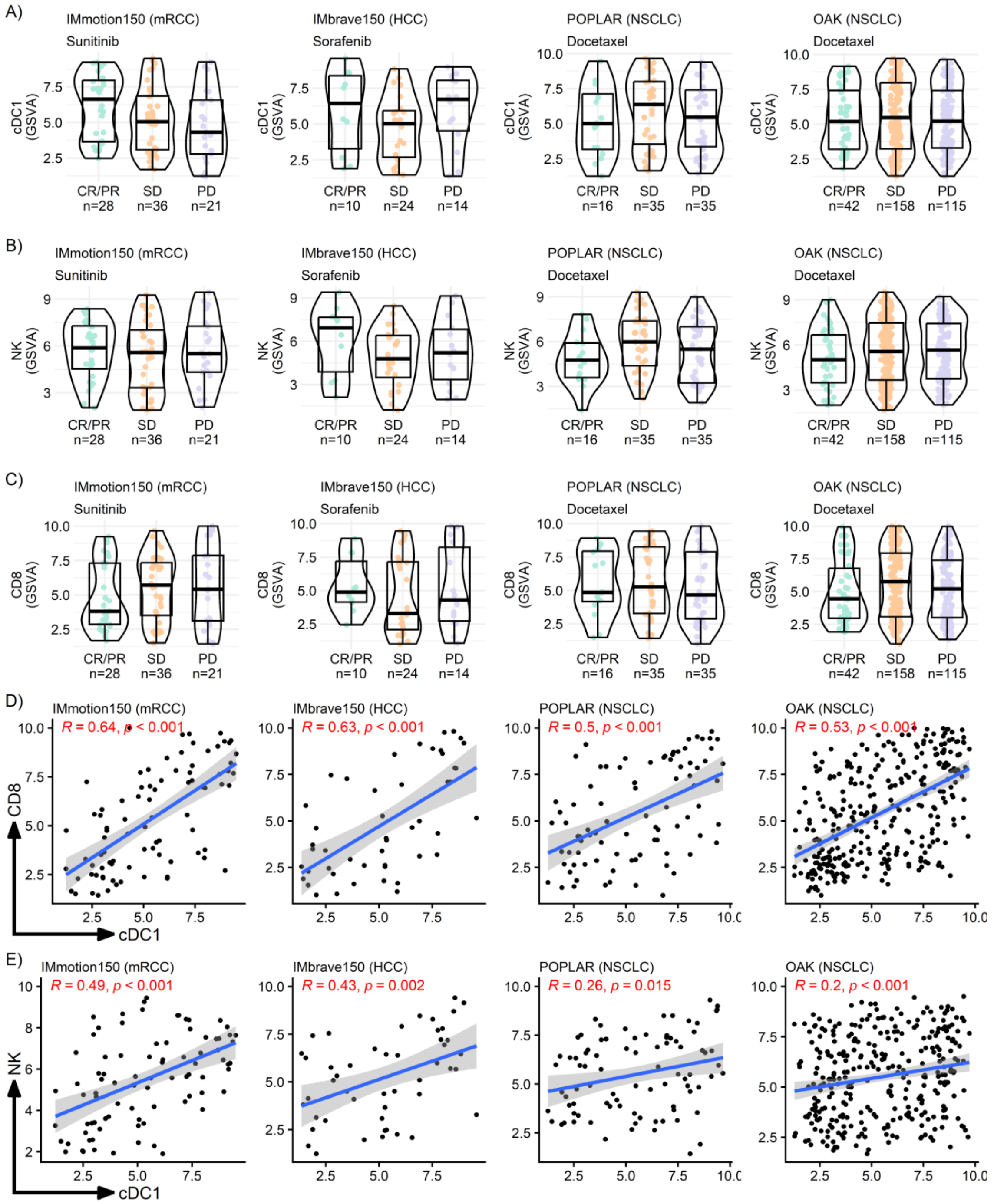

Supplementary figure 1 Non immunotherapy control arms in the indicated clinical trials show a lack of association of the cDC1 signature with clinical benefit whilst the correlations with CD8 and NK infiltration are preserved. (**A** to **C**) Data as in figure 1 representing the gene signatures for cDC1, CD8 and NK cells in patients classified according to clinical benefit from arms that did not receive immunotherapy. (**D** to **E**) Represent the statistical correlations (according to Pearson correlation coeffiecient) of

gene expression in patients’ tumor samples of the cDC1 gene signature with the NK and CD8 signatures. The p values are calculated according to linear regression. Shaded area represents the confidence intervals for each linear regression line.

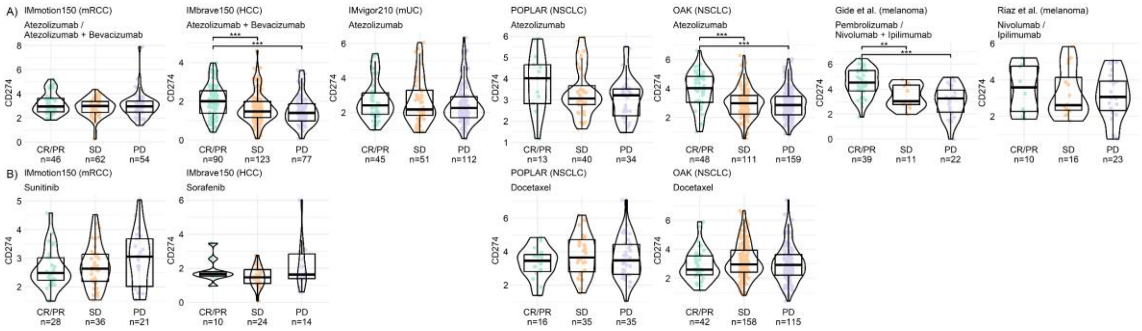

Supplementary figure 2. Studies on the associations of PDL1 mRNA expression (CD274) with clinical outcome. Data represents associations of PDL1 in some but not all the trials testing checkpoint inhibitors **(A)** and in no series of those patients treated with targeted therapy or taxane chemotherapy in the corresponding control arms **(B)**. Asterisks indicate p values for comparisons according to Wilcoxon significance tests: * <0.05, **<0.01, ***<0.001.

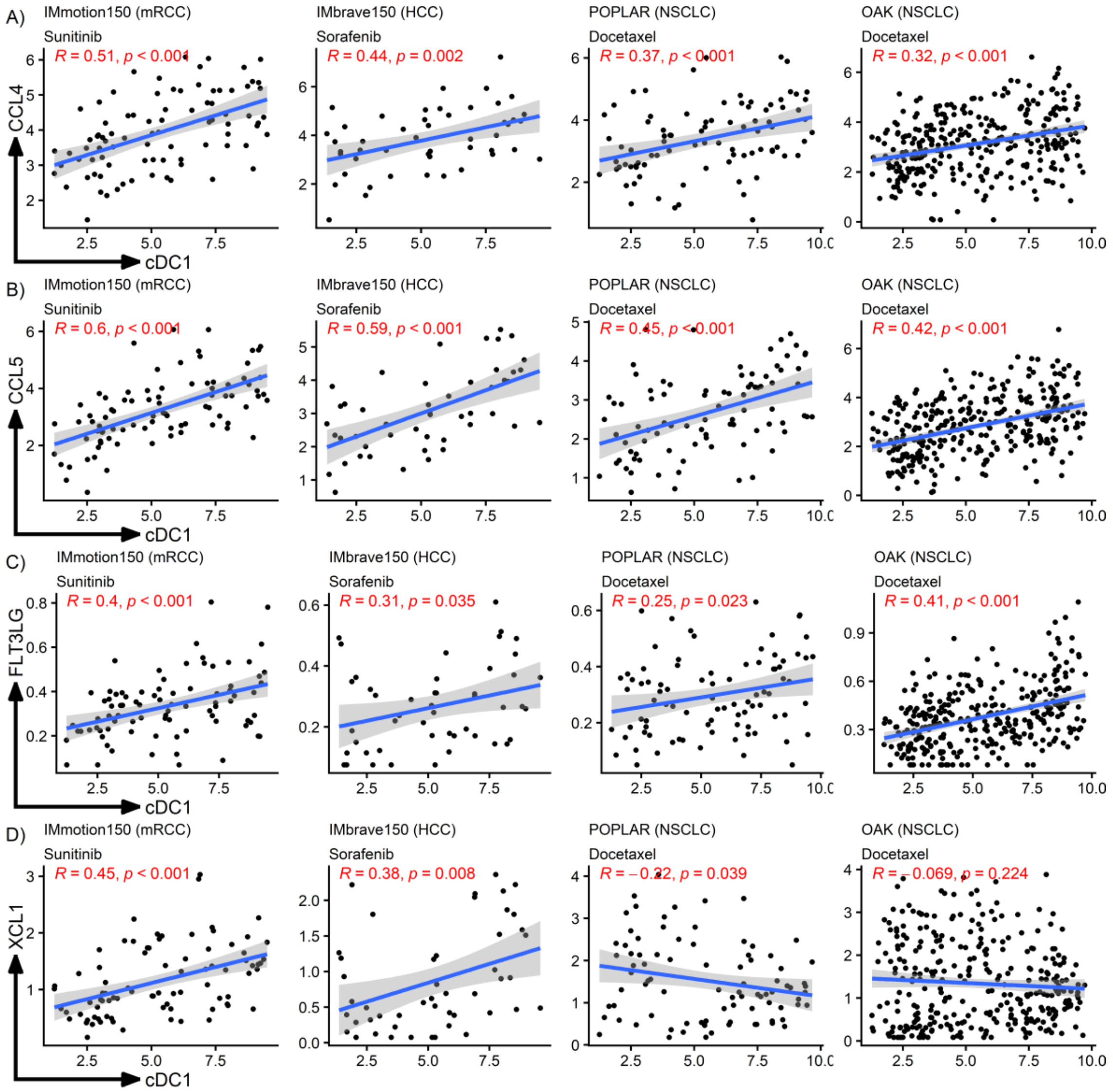

Supplementary figure 3 The transcripts for CCL4, CCL5 and FLT3LG correlate with cDC1 also in the series of patients treated with targeted therapy or chemotherapy across the trials. (**A** to **C**) Data as in figure 2 in which the indicated transcripts in tumors from patients treated in the non-immunotherapy arms across trials were studied for the association with the cDC1 signature as indicated. In (**D**) a variable association with XCL1 was noted. All correlation indexes refer to Pearson correlation. The p values are calculated according to linear regression. Shaded area represents the confidence intervals for each linear regression line.

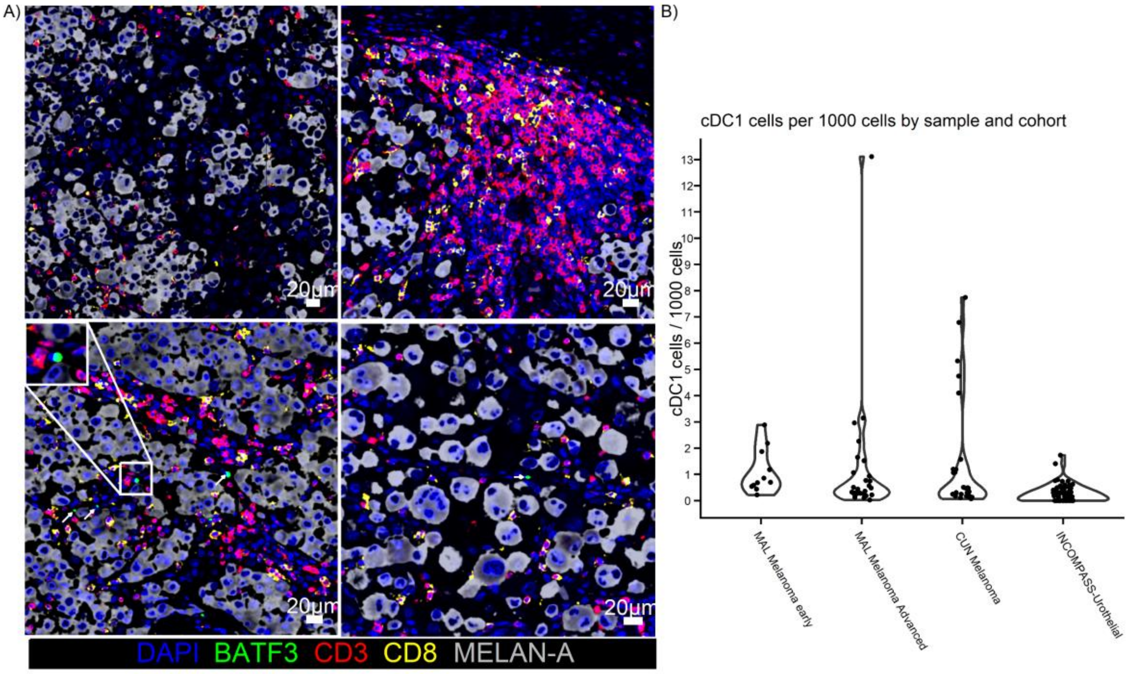

Supplementary figure 4. Representative microphotographs as those analyzed in figure 4.

A) Four representative microphotographs of different patterns of cDC1 and T-cell lymphocyte tumor infiltration from the CUN melanoma cohort. Upper left: Area devoid of cDC1, and only occasional T-cells. Upper right: Area with a high-density T cell infiltration but absence of cDC1 cells. Lower left: Area with occasional BATF3+ cDC1 cells in close proximity to T cells. Inset and white arrows indicate BATF3+ cDC1 cells. Lower right: Area with an isolated BATF3+ cDC1 cell (white arrow) and very few infiltrating T cells. B) Number of cDC1 BATF3+ cells per 1000 cells analyzed across cohorts.

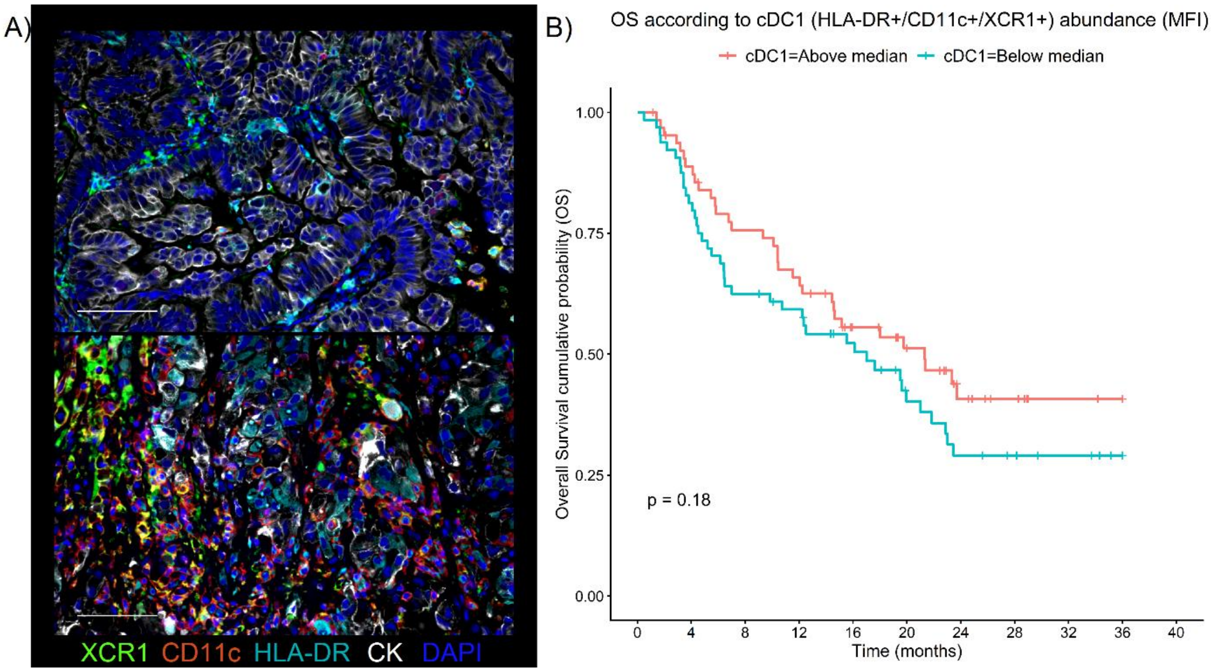

Supplementary figure 5. Multiplex immunofluorescence analyses of NSCLC samples from patients treated with immunotherapy show a positive association between cDC1 density and Overall Survival. A) Representative microphotographs of NSCLC samples demonstrating low and high density of cDC1. Scale bar = 100 µm. B) Kaplan-Meier plot demonstrating association between cDC1 density and overall survival.

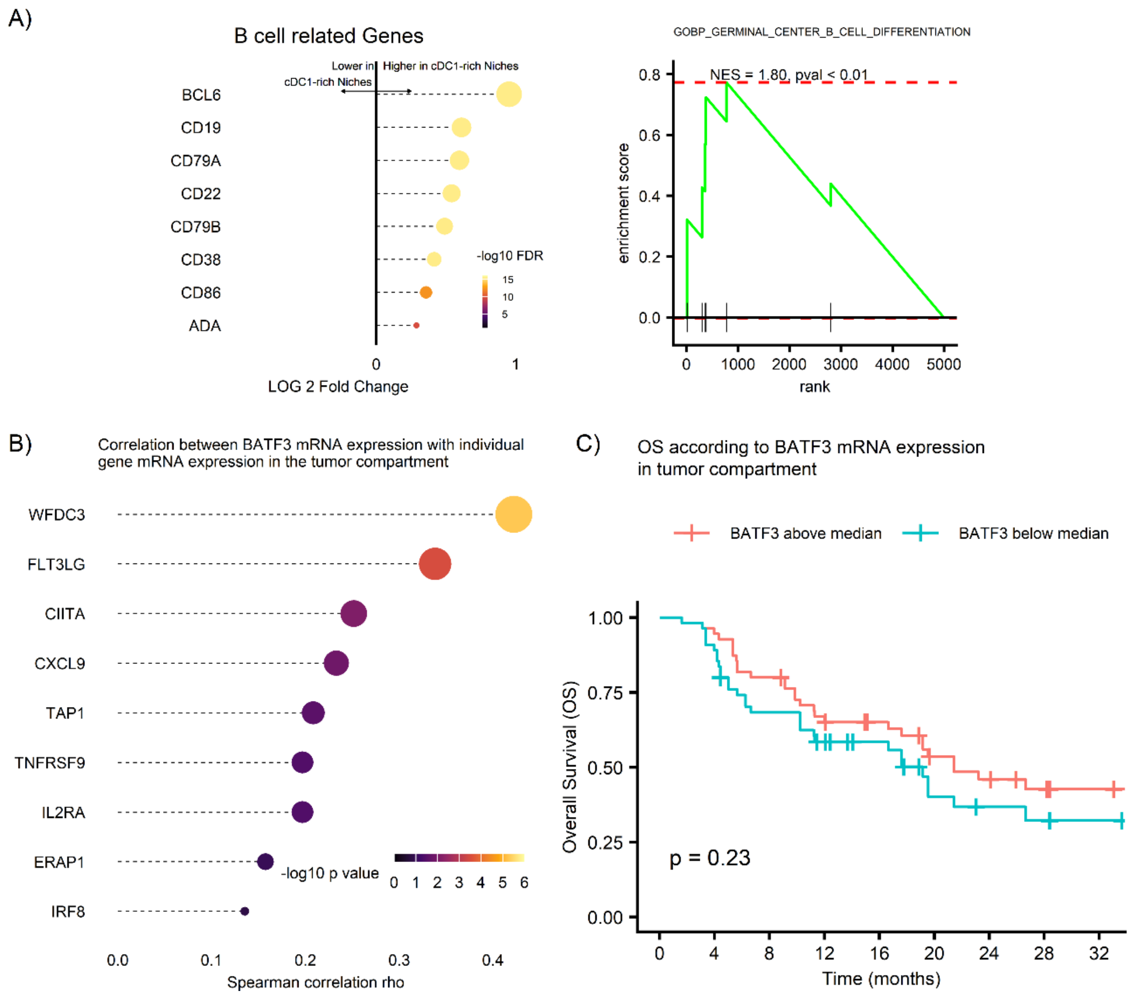

Supplementary figure 6. cDC1 niche analysis reveals increased B lymphocyte activity in cDC1 rich samples. GeoMX spatial transcriptomic analysis of NSCLC samples from immunotherapy treated patients demonstrates an association of cDC1 specific transcript spatial abundance with immunoregulatory genes and survival. A) Niche results illustrating selected B lymphocyte associated transcripts that are differentially overexpressed in cDC1 rich niche. GSEA enrichment plot revealing upregulation of germinal center associated pathway. In the left panel, point sizes reflect fold change values. B) Spearman correlation analysis between BATF3 mRNA expression in the tumor compartment and key immune function associated transcripts. C) Kaplan-Meier analysis revealing a positive non-significant association between BATF3 mRNA expression in the tumor compartment and overall survival.

Supplementary table 1

Cohort	Tumor type	Tumor stage	Sample Size	Treatment received	Endpoint	Sample Obtention	Sample type
CUN Melanoma	Melanoma	Advanced/Metastatic	30	Nivolumab±Ipilimumab	Radiologic response at 12 months per RECIST 1.1 criteria	Pre-treatment	Whole Slide
MAL Melanoma Advanced	Melanoma	Advanced/Metastatic	20	Nivolumab / Pembrolizumab+TKI / Nivolumab+Ipilimumab	Progression before 3 months / No progression for 12 months	Pre-treatment	Whole Slide
MAL Melanoma Early	Melanoma	Early stage	11	Nivolumab / Pembrolizumab	Progression before 3 months / No progression for 12 months	Pre-treatment	Whole Slide
INCOMPASS-Urothelial	Urothelial carcinoma	Advanced/Metastatic	34	Atezolizumab	Best radiologic response per RECIST 1.1 criteria	Pre-treatment	Tissue Microarray
YALE-NSCLC	NSCLC	Advanced/Metastatic	130	Atezolizumab / Pembrolizumab / Nivolumab / Nivolumab+Ipilimumab	Overall Survival	Pre-treatment	Tissue Microarray

FU: Follow-up
